# Population Pharmacokinetic Model of Linezolid and Probability of Target Attainment in Patients with COVID-19-Associated Acute Respiratory Distress Syndrome on Veno-Venous Extracorporeal Membrane Oxygenation—A Step toward Correct Dosing

**DOI:** 10.3390/pharmaceutics16020253

**Published:** 2024-02-08

**Authors:** Dragana Milaković, Tijana Kovačević, Pedja Kovačević, Vedrana Barišić, Sanja Avram, Saša Dragić, Biljana Zlojutro, Danica Momčičević, Branislava Miljković, Katarina Vučićević

**Affiliations:** 1 Department of Nuclear Medicine and Thyroid Gland Diseases, University Clinical Centre of the Republic of Srpska, 78000 Banja Luka, Bosnia and Herzegovina; 2 Pharmacy Department, University Clinical Centre of the Republic of Srpska, 78000 Banja Luka, Bosnia and Herzegovina; 3 Faculty of Medicine, University of Banja Luka, 78000 Banja Luka, Bosnia and Herzegovina; pedja.kovacevic@kc-bl.com (P.K.);; 4 Medical Intensive Care Unit, University Clinical Centre of the Republic of Srpska, 78000 Banja Luka, Bosnia and Herzegovina; 5 Institute of Laboratory Diagnostic, University Clinical Centre of the Republic of Srpska, 78000 Banja Luka, Bosnia and Herzegovina; 6 Department of Pharmacokinetics and Clinical Pharmacy, University of Belgrade-Faculty of Pharmacy, 11221 Belgrade, Serbia

**Keywords:** COVID-19, ECMO, linezolid, population pharmacokinetics, dosing, PTA

## Abstract

During veno-venous extracorporeal membrane oxygenation (vv ECMO) therapy, antimicrobial drugs are frequently used, and appropriate dosing is challenging due to there being limited data to support the dosage. Linezolid is effective against multidrug-resistant Gram-positive pathogens frequently isolated in ECMO patients. In total, 53 steady-state linezolid levels were obtained following 600 mg intravenous (IV) injections every 8 h, and these were used to develop a population pharmacokinetic (PopPK) model in patients with COVID-19-associated acute respiratory distress syndrome (CARDS) on vv ECMO. The data were analyzed using a nonlinear mixed-effects modelling approach. Monte Carlo simulation generated 5000 patients’ individual PK parameters and corresponding concentration–time profiles using the PopPK model, following the administration of 600 mg/8 h (a higher-than-standard dosing) and 600 mg/12 h (standard). The probabilities of pharmacokinetic/pharmacodynamic (PK/PD) target attainment (PTA) and the cumulative fraction of responses (CFR) for three pathogens were calculated and compared between the two dosing scenarios. Linezolid 600 mg/8 h was predicted to achieve greater than or equal to 85%T*f*>MIC in at least 90% of the patients with CARDS on vv ECMO compared to only approximately two thirds of the patients after dosing every 12 h at a minimal inhibitory concentration (MIC) of 2 mg/L. In addition, for the same MIC, *f*AUC_24_/MIC ≥ 80 was achieved in almost three times the number of patients following an 8-h versus a 12-h interval. PopPK simulation predicted that a significantly higher proportion of the patients with CARDS on vv ECMO would achieve the PK/PD targets following the 8-h dosing interval compared to standard linezolid dosing. Nevertheless, the safety concern, in particular, for thrombocytopenia, with higher-than-standard linezolid dosage is reasonable, and consequently, monitoring is essential.

## 1. Introduction

Millions of people worldwide diagnosed with COVID-19 developed pneumonia from the beginning of 2020 until the end of 2021, and 5% of them developed a critical illness related to acute respiratory distress syndrome (ARDS), with an unacceptably high mortality rate (of at least 45%), especially in low-resource settings [1,2]. COVID-19-associated ARDS (CARDS) is connected with the extended length of both intensive care unit (ICU) and hospital stays, with prolonged mechanical ventilation, and in some cases, with veno-venous extracorporeal membrane oxygenation (vv ECMO) as a last option for advanced life support [3]. During the COVID-19 pandemic, the frequent use of vv ECMO in adult patients was reported [4,5]. While vv ECMO provides respiratory support, allowing the heart to pump blood, veno-arterial ECMO (va ECMO) provides cardio-respiratory support, where the machine oxygenates and pumps the blood. Vv ECMO as a mechanical life support requires an invasive procedure (cannulation), i.e., the precise insertion of a large-diameter cannula; however, this population is prone to hospital-acquired infections [6]. Choosing the correct antimicrobial therapy is important in reducing the number of nosocomial infections, as well as the mortality rate [7]. The pathophysiology of critically ill patients is a unique entity characterized by dramatically altered drugs’ pharmacokinetics (PK) and pharmacodynamics (PD) compared with those of the noncritically ill patients, which ultimately affects the therapeutic outcomes [8,9]. The complex system and mechanism of action of vv ECMO can alter the antimicrobial PK, which may result in therapeutic failure or toxicity. A life support procedure with vv ECMO can affect the drug’s PK in three ways: drug sequestration in the vv ECMO circuit, the volume of distribution (Vd) increases, or an alteration in drug clearance (CL) [10,11,12]. In general, the data regarding the in vivo linezolid characteristics associated with vv ECMO treatment are scarce, and presently, there are limited data to support using a therapeutic dosage of antibiotics to treat this group of patients. As a side note, we expect to observe no differences with respect to the modality of the ECMO (vv or va) procedure on the drugs’ PK, including linezolid. However, variations between the patients are based on the ECMO circuit characteristics and the patient’s underlying health condition. Therapeutic drug monitoring (TDM) is useful in these circumstances, but it should be noted that it is not always possible. Knowledge of the physicochemical properties of the drug, the availability of published data, and the application of TDM can be helpful for choosing the best dosage regimen of an antibiotic [11].

Linezolid was the oxazolidinone antibiotic to be licensed; it is a synthetic antimicrobial with activity against most Gram-positive bacteria and some species of Mycobacteria [13,14]. Linezolid has hydrophilic and moderately lipophilic characteristics, a plasma protein binding of 31%, and a Vd about 40–50 L, which approximates to total body water. Excretion occurs predominantly via renal elimination; under steady-state conditions, 30% of the total dose appears in urine as linezolid, and ~50% appears as metabolites [15,16,17,18]. The antimicrobial efficacy of linezolid is best described by the relationship between the percentage of time that the free linezolid concentration remains above the minimum inhibitory concentration (MIC) (%T*f*>MIC: >85%) and the area under the free concentration–time curve for 24 h divided by the MIC (*f*AUC_24_/MIC: 80–120) [17,19]. The recommended target range for linezolid trough concentrations is from 2 to 8 mg/L. However, it is worth noting that trough concentrations of 6.53 mg/L and 9.96 mg/L are associated with 50% and 90% probabilities of thrombocytopenia during prolonged treatment, respectively [20]. Because of the physicochemical and PK characteristics of this drug, routine TDM and the adjustment of the dosing regimen may not be necessary in most cases. However, the recent studies suggest that linezolid TDM may be beneficial for dosing adjustments in ICUs or in some other settings [21,22,23].

Interindividual variability in drug exposure and the influence of pathophysiological changes on the PK characteristics can lead to linezolid overexposure or underexposure, resulting adverse reactions or therapeutic failure, respectively. In up to 30% of critically ill patients, the linezolid trough levels were outside the target reference range, both above and below, following a standard dosing regimen [14,19,21,22]. Hence, linezolid concentrations show high-level interindividual variability in critically ill patients. The inherent physiological changes associated with ECMO and critical illness additionally influence the PK changes of a drug, making exact prediction more difficult [7].

The standard intravenous (IV) linezolid dosage is 600 mg every 12 h [15]. However, there are limited data on the dosing recommendations of linezolid to ensure adequate efficacy and safety during ECMO therapy. Nevertheless, the recent expert consensus supports the optimization of the linezolid dosage for patients on ECMO based on TDM and, perhaps, the MIC value [24]. The hospital in this study administered a higher-than-standard intravenous linezolid dosage of 600 mg IV every 8 h during vv ECMO. After an exhaustive review, we found only three reports/manuscripts documented similar cases [25,26,27], while in two, the linezolid daily dose was 1800 mg, which was administered via continuous infusion [27] or divided into three doses [26].

Nonlinear mixed-effects modeling is a statistical approach that is particularly valuable in addressing the variability in drug concentrations among patients, while estimating the individual and population (Pop) model parameters. Various PopPK models for linezolid exist, with some encompassing populations of critically ill patients, as detailed in a comprehensive review manuscript by Bandin-Vilar et al. [28]. Combining these models with the MIC is fundamental for optimizing the dosing regimen for specific patient subpopulations. Furthermore, the integration of the Bayesian approach with PopPK models and coupled with TDM is the cornerstone of the model-informed precision dosing (MIPD) concept. Hence, MIPD facilitates the more precise attainment of PK/PD targets in individual patient, maximizing the efficacy and minimizing the probability of antibiotic toxicity [29,30,31].

The aim of this clinical study was to describe the concentration–time profiles of the linezolid in patients with CARDS on vv ECMO receiving 600 mg of linezolid via 30-min infusions every 8 h and to develop a PopPK model. Moreover, we assessed the probability of attaining the antimicrobial efficacy targets utilizing the developed PopPK model after administering standard (600 mg every 12 h) and higher-than-standard dosing regimens through Monte Carlo simulations.

## 2. Materials and Methods

### 2.1. Settings

A prospective, observational, single-center PK study was conducted between 1 January and 31 December 2021, in the 28-bed medical intensive care unit (MICU) of a university-affiliated, tertiary care hospital: the University Clinical Centre of the Republic of Srpska (UCC RS), Banja Luka. The MICU serves as a referral center for the Republic of Srpska, with approximately 1,000,000 inhabitants, and is currently the most advanced multidisciplinary MICU in Bosnia and Herzegovina (low-resource setting), which is a post-conflict country undergoing transition.

### 2.2. Study Population

Patients with CARDS (lung injury/Murray/score ≥ 3, or uncompensated hypercapnic acidosis with pH < 7.2) treated with vv ECMO who were receiving linezolid during extracorporeal life support met the inclusion criteria. Hence, all the patients were confirmed to have COVID-19 using a reverse transcription–polymerase chain reaction (RT-PCR) from nasopharyngeal swab specimens and respiratory secretions upon hospital admission. The exclusion criteria were being age <18 years, having a known allergy to linezolid, being pregnant, and having undergone therapeutic plasma exchange in the proceeding 24 h or renal replacement therapy (RRT).

### 2.3. Extracorporeal Membrane Oxygenation System

All the patients were treated using an extracorporeal life support machine called Cardiohelp™ (Maquet). A CARDIOHELP ECMO pump (Maquet Getinge Group CARDIOHELP-I REF70104-8012) and circuit (Maquet HLS Set Advanced 7.0, HLS 7050 USA, 701052794) were used. Cannulation was performed using Maquet Arterial and Venous Cannulas (PAL 1523, PVS 1938, and PVL 2155). The disposable pack for the Cardiohelp™ system is all-inclusive and contains all the supplies necessary for ECMO support. Briefly, the kit consists of pre-connected arterial and venous 3/8″ tubing and an oxygenator/heat exchanger with an integrated blood pump. All the temperature, pressure, and blood gas measurement technologies are integrated into the oxygenator/centrifugal blood pump module, and the values are displayed on the Cardiohelp™ drive unit. The standard circuit consists of an X-coated custom 3/8″ tubing pack (Terumo, Ann Arbor, MI, USA), a Quadrox-D oxygenator (Maquet Cardiovascular, Bridgewater, NJ, USA), and a CentriMag blood pump (Thoratec Corp., Pleasanton, CA, USA). For this circuit, a CDI-500 (Terumo Medical Corp., Ann Arbor, MI, USA) in-line blood gas analyzer was incorporated to measure the blood gases and electrolytes on the arterial side (via intra-circuit shunt), and a venous cuvette was used to measure the hemoglobin, hematocrit, and venous oxygen saturation levels. The circuit pressures were monitored at the inlet and outlet of the oxygenator and were transduced to the CentriMag console.

### 2.4. Linezolid Dosing and Administration

Linezolid, as a solution for infusion, was administered IV via 30-min infusions at a dose of 600 mg every 8 h, according to the local hospital ICU ECMO protocol. This off-label dosing regimen was locally adopted due to several factors, including antibiotic-resistant organisms within the hospital, the PK considerations in this specific patient population, and our hospital’s experience with linezolid therapy in critically ill ECMO patients, as supported by the expert consensus that the linezolid dosing regimen need to be adjusted based on TDM for patients on ECMO [24].

### 2.5. Study Protocol and Linezolid Assay

PK sampling was performed at a steady-state linezolid concentration during ECMO therapy. To ensure a steady-state concentration, at least six doses were administered before sampling. Blood was drawn into 6 mL clot activator tubes from an existing arterial or venous line at the following timepoints: pre-dose and 30, 60, 120, 240, and 360 min after the start of the infusion. The blood samples were centrifuged at 3000× *g* for 15 min, and the sera were kept frozen at −80 °C until analysis. Linezolid concentration was measured with the DxC 700 AU analyzer (Beckman Coulter Inc., Brea, CA, USA) using the homogeneous enzyme immunoassay kit ARK™ Linezolid Assay (ARK Diagnostics, Inc., Fremont, CA, USA) with measurement ranges from 0.75 to 30 μg/mL.

### 2.6. Data

The linezolid dosing data included the following: the date of the first dose, the start and end of infusion, the number of doses received before sampling, and the exact timing of each linezolid sample. Detailed clinical and demographic data were collected for all the patients, including the following: the admission diagnosis; date of admission to the MICU; comorbidities; age; sex; body weight; height; therapy after admission to the ward; body temperature; pulse; mean arterial pressure; serum levels of albumin, liver enzymes, bilirubin, C-reactive protein, and D-dimer; thrombocyte and serum creatinine counts; urea; 24 h urine output; Simplified Acute Physiology Score (SAPS) II on admission day; Sequential Organ Failure Assessment (SOFA) on PK sampling day; start of ECMO therapy; and ECMO parameters on the PK sampling day.

### 2.7. Population Pharmacokinetics (PopPK) Analysis

The linezolid PK data were analyzed using a nonlinear mixed-effects modelling approach implemented with NONMEM^®^ software (version 7.5). The mono- and bi-exponential decays in concentration over time were tested, and the corresponding parameters were estimated. In addition, the between-subject variability and residual variability were estimated using exponential and proportional or combined models, respectively. The relationships between the PK parameters and the demographic, treatment, or clinical factors (covariates) were examined using an automated covariate model building procedure with standard criteria for the inclusion or elimination of the covariates within and from the model (p_forward_ < 0.05 and p_backward_ > 0.01, respectively). The critical models were checked for adequacy using numerical and graphical evaluations, and the final model was validated. A visual predictive check (VPC) was performed with the Pearl-speaks-NONMEM module (PsN, version 5.2.6) based on 1000 simulations. Bootstrap 95% confidence intervals for the estimated parameters were evaluated [32]. For each patient, the individual AUC_24_ was calculated based on the dosing regimen and estimated individual CL.

### 2.8. Monte Carlo Simulation and Probability of Target Attainment

The developed linezolid PopPK model was used to simulate each of the 5000 patients’ set of PK parameters using random sampling from the predefined parameter distribution. Individual PK parameters were generated for each simulated patient. Monte Carlo simulation was performed using NONMEM^®^ software (version 7.5). Three PK/PD targets (85%T*f*>MIC, *f*AUC_24_/MIC > 80, and *f*AUC_24_/MIC > 100) were selected for analysis, as previously described. For this calculation, the free concentration and AUC were calculated based on the literature value for the fraction unbound (*f_ub_*) of 0.69 [15] and two dosing schedules of linezolid. The probability of target attainment (PTA) was calculated for two different dosing regimens at 30 min of linezolid infusion: 600 mg every 12 h and 600 mg every 8 h for a range of minimal inhibitory concentrations (MICs). A PTA of minimum 90% was considered to be desirable. These data were evaluated against the susceptibility data. By comparing the PTA against the MIC distributions from the European Committee on Antimicrobial Susceptibility Testing (EUCAST) data for Gram-positive isolates (*Enterococcus faecium*, *Staphylococcus aureus*, and *Streptococcus pneumoniae*) [33], the cumulative fraction of responses (CFRs) were calculated using the following equation:

(1) $$FR=\overset{n}{\underset{i=1}{\sum }}PTA_{i}\cdot F_{i}$$

where PTA_i_ refers to each MIC, and F_i_ represents the fraction of the population of microorganisms in each MIC category [34]. A CFR of a minimum 90% was defined as acceptable. The graphical processing of the data and NONMEM^®^ output were performed with R programming language (version 4.1.3, R Foundation for Statistical Computing, Vienna, Austria) in RStudio (desktop version 1.4.1717) using various packages.

## 3. Results

PK sampling was performed on eleven patients; however, two patients were excluded from the analysis due to dialysis and transfusion during the vv ECMO and linezolid treatments. Based on the measured linezolid concentration at each time point, these two patients had, in total, five concentrations below the lower limit of quantification (BLLOQ). One of them was on continuous veno-venous hemodialysis (CVVHD) in combination with treatment using a CytoSorb^®^ device, while the other patient received a blood transfusion during the sampling period. In line with the previous results, we also observed decreased linezolid exposure in these two patients compared to that of the other patients in the study. To minimize the confounding effects of these variables on the linezolid PK and ensure that the patient characteristics were relatively homogeneous, these two patients were excluded from PopPK analysis, as defined by the exclusion criteria. The relevant demographics and clinical characteristics of the patients included in the analysis are reported in Table 1.

**Figure 1:**
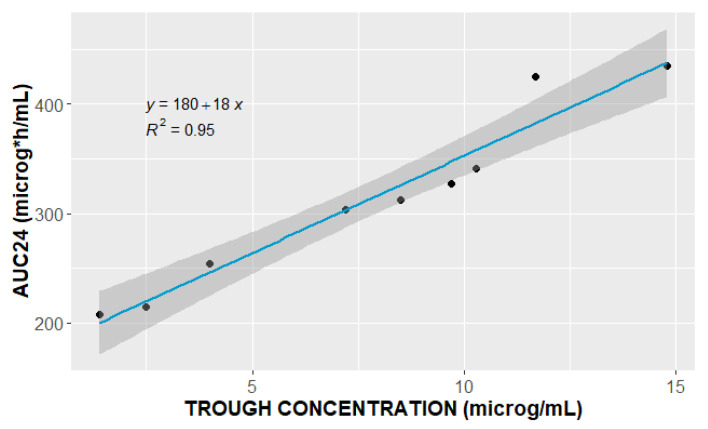
Relationship between observed linezolid trough concentration (μg/mL) and area under the curve over 24 h (AUC_24_, μg·h/mL).

Consequently, 53 PK samples from nine patients were used for PopPK analysis. The PK sample at 0.5 h in ID8 was not obtained at the designated time point due to a protocol error. All the patients included in the analysis, except for one, had trough levels above 2 μg/mL, while the peak levels were less than 25.0 μg/mL. The median linezolid trough and peak concentrations were 8.5 (range: 1.4–14.8) μg/mL, and 22.26 (range: 20.8–25.0) μg/mL, respectively. The observed trough levels were highly correlated (R^2^ = 0.95) with the patients’ daily AUCs (Figure 1).

**Figure 2:**
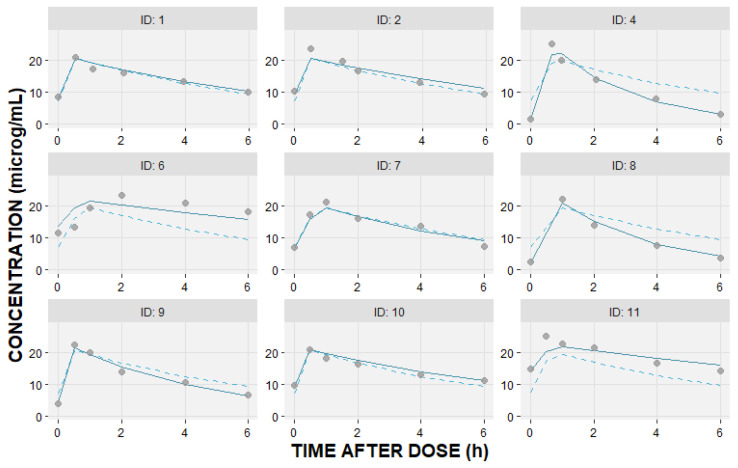
Individual linezolid observed—DV (circles); individual predicted—IPRED (solid line); and population predicted—PRED (dashed line) concentrations (μg/mL) versus time after dosing (hours) based on the fitted one-compartment pharmacokinetic model.

A one-compartment model with first-order elimination best described pooled concentration–time data. Figure 2 shows the individual measured levels and population model that best fitted the data, together with the individual predicted concentration–time profiles.

The individual observed trough and peak linezolid concentrations, together with the estimated total area under the curve over 24 h (AUC_24_) and half-life values, are given in Table 2. 

The interindividual variability in CL and Vd can be explained using an exponential model with covariance among the parameters, whereas the residual variability was modelled as a proportional error model. The covariates were systematically examined. However, probably because of the small sample size, none of the covariates showed a significant effect on the PK parameters. The value of objective function (OFV) decreased by 2.04 units when the ECMO centrifugal pump speed, as a covariate, was included in the base model and tested against it. However, a decrease of at least 3.84 points for one degree of freedom was considered as a significant improvement of the fit with a *p*-value of <0.05. The final parameter estimates and their bootstrap 95% confidence intervals are summarized in Table 3. The parameters were estimated with an uncertainty ranging from 8% for CL to 19% for the interindividual variability in Vd (Table 3).

**Figure 3:**
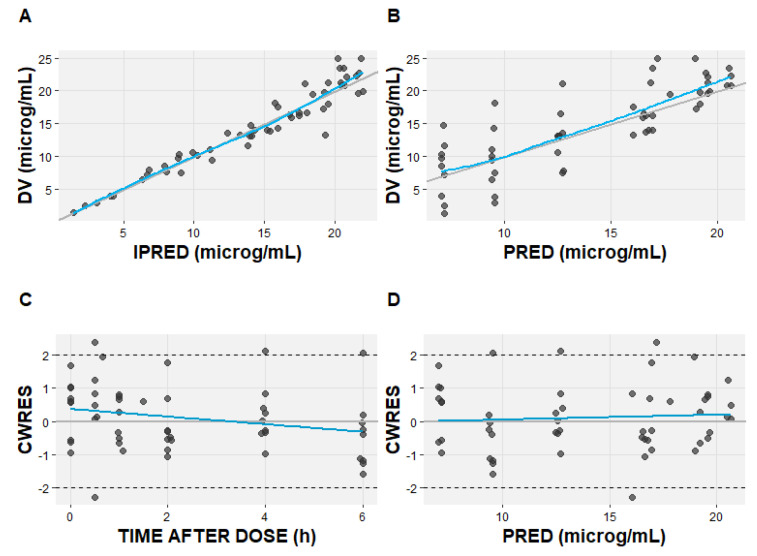
The goodness-of-fit plots of the final linezolid pharmacokinetic model. (**A**) Observed (DV) versus individual predicted concentrations (IPRED); (**B**) observed (DV) versus population predicted concentrations (PRED); (**C**) conditional weighted residual (CWRES) versus time after dose; (**D**) conditional weighted residual versus population predicted concentration (PRED). The solid blue lines represent the line of identity; the grey line represents linear regression.

**Figure 4:**
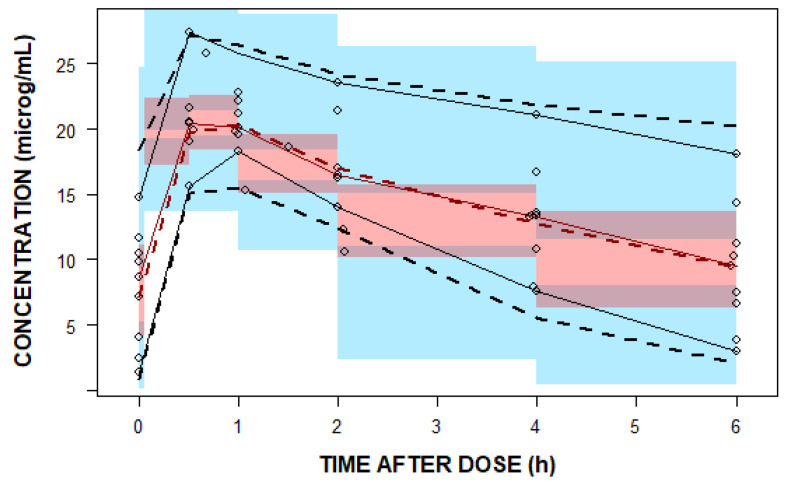
A visual predicted check (VPC) plot of the final population pharmacokinetic (PopPK) model. The open black circles represent the observed linezolid concentrations. The solid red line represents the 50th percentile and solid black lines represent the 5th and 95th percentiles of the observations. The semi-transparent red shaded area represents the simulation-based 95% confidence interval (CI) for the median, while the semi-transparent blue fields represent the 95% CI around the 5th and 95th percentiles of the predicted data. The dashed red and black lines represent the 50th and the 5th and 90th percentiles of the predictions, respectively.

One thousand bootstrap runs indicated that the model parameters were sufficiently robust (Table 3). The standard goodness-of-fit plots indicate good agreement among the observed and predicted linezolid concentrations (Figure 3). The visual predicted check (VPC) plot shows the good predictive performance of the final model (Figure 4).

**Figure 5:**
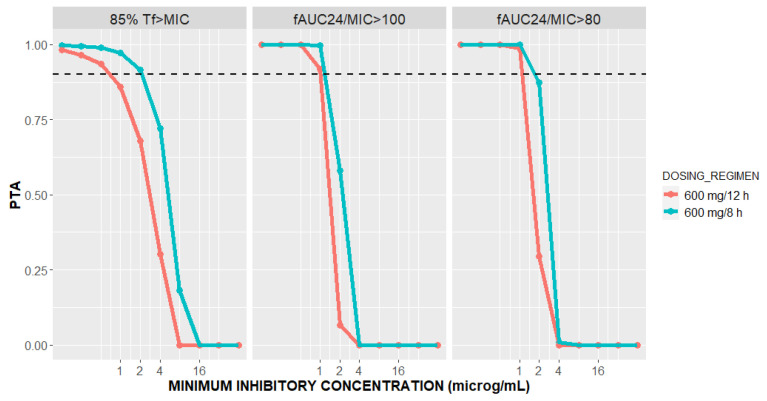
Probability of targets attainment (PTA) for 85%T*f*>MIC and *f*AUC_24_/MIC of 100 and 80 versus minimal inhibitory concentration (MIC) for patients with CARDS on vv ECMO at two dosing regimens of linezolid.

Based on the PopPK model, we simulated the PK profiles of 5000 patients according to two dosing schedules via 30-min linezolid infusions of 600 mg every 8 and every 12 h, and the corresponding individual dose-dependent daily AUCs were calculated. Using the following PK/PD targets, 85%T*f*>MIC and *f*AUC_24_/MIC of 80 and 100, the PTAs for two linezolid dosing regimens were calculated and are given in Figure 5.

**Figure 6:**
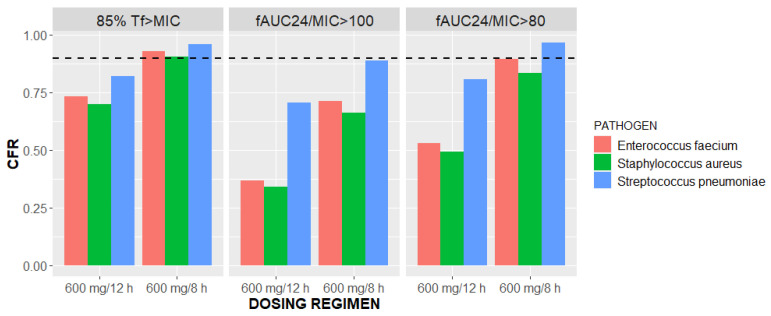
Cumulative fraction of responses (CFRs) versus linezolid dosing regimens of 600 mg/12 h and 600 mg/8 h stratified by three pathogens and three PK/PD targets: 85%T*f*>MIC and *f*AUC_24_/MIC of 100 and 80.

The CFRs were calculated based on simulated PTAs and EUCAST MIC distributions for three selected pathogens for each of the PK/PD targets and two dosing regimens (Figure 6).

## 4. Discussion

A higher-than-standard dose of linezolid may be required to achieve the PK/PD target(s) against the less-susceptible pathogens (*Enterococcus faecium*, *Staphylococcus aureus*, and *Streptococcus pneumoniae*) in critically ill patients with COVID-19 on vv ECMO therapy. To the best of our knowledge, this is the first study to investigate linezolid PK/PD in the patients with CARDS on vv ECMO therapy. In addition to the above main findings, this study supports linezolid dosing of 600 mg every 8 h, which is in compliance with the local hospital’s ICU vv ECMO protocol. Moreover, our findings highlight the importance of a therapeutically adequate linezolid dosage among patients on vv ECMO and support the consensus of the expert panel that critically ill patients on vv ECMO should receive a linezolid dosage of 600 mg every 8 h when the MIC = 2 mg/L [24].

Differing from the other studies found, which involved the administration of linezolid at a dosage of 600 mg every 12 h via both oral and IV routes and achieved trough concentrations within the predefined range in approximately 50–60% of patients [23,35], our study observed that only four of our patients achieved trough concentrations of 2–8 μg/mL (Table 2). Pea et al. assessed the linezolid TDM data from 10 years of experience with a large patient population, including around 20% of ICU patients and a specified desired linezolid trough from 2 to 7 μg/mL [23]. This variation in our findings compared to those in other publications was anticipated and can be attributed to the variations in dosing regimens. Specifically, an 8-h dosing interval yields elevated pre-dose concentrations when compared to a 12-h dosing regimen. The administration of antibiotics, including linezolid, during ECMO poses a challenge in clinical practice, as its impact on drugs’ Vd and CL can be significant [10,11]. The data on linezolid PK in vv ECMO patients are limited, but there are a few reports advocating for a daily linezolid dose of 1800 mg [26,27]. Our results approximate the findings of the study by Kühn et al. [27], in which linezolid was administered via continuous infusion, and the median level was 8.6 (IQR: 5–10.5) μg/mL in ECMO patients, while the targeted value was 6.5–12 μg/mL. In contrast, another case report presented an ECMO patient after a right lung transplantation whose linezolid trough level was 0.35 μg/mL after 600 mg/8 h dosing [26]. The authors pointed out that this patient had significant hypoalbuminemia that may have contributed to an increase in Vd and CL, and thus, to such a low drug level [26]. In our study, all the patients had normal albumin levels (Table 1). Although our study included only vv ECMO patients, the linezolid trough levels were expected to be lower than those in the non-ECMO patients; Kühn et al. observed a tendency toward lower concentrations, but without a statistically significant difference [27]. Based on the previous reports, vv ECMO may lead to hemodilution [7], and certain drugs may adsorb to the materials used in vv ECMO circuits, resulting in decreased antibiotic concentrations in the patients’ bloodstream. Factors such as hemodilution and changes in pH during vv ECMO can influence the protein binding of antibiotics [11]. Vv ECMO can also affect a patient’s fluid balance, subsequently impacting their renal function and, consequently, the excretion of antibiotics, thus influencing their PK profile [11,12].

As shown in Figure 1, our results indicate high-level correlation (R^2^ = 0.95) between the linezolid trough concentration and AUC_24_, supporting the idea of sparse sampling and use of trough levels as a surrogate for AUC_24_ during routine clinical TDM, as previously suggested [36].

Of note, one of the excluded patients in this study had a linezolid trough level BLLOQ and a shorter half-life during concurrent CVVHD in combination with the CytoSorb^®^ treatment, suggesting their combined effect on the drug’s PK profile.

It has been suggested that linezolid concentrations above 8 μg/mL are correlated with side effects/toxicity known as thrombocytopenia [21,37]. In five patients included in our study, the trough concentration exceeded the reported threshold, while a platelet count of less than 150 × 10^9^ cells/L on the PK sampling day was present in seven patients. In patients with CARDS on vv ECMO, thrombocytopenia may occur due to not only linezolid, but also a combination of several associated factors including the severity of infection, the use of vv ECMO, and the baseline platelet count.

The one-compartment model best fitted the concentration–time linezolid data. None of the tested covariates showed a significant effect on the PK parameters, probably due to the small sample size (Table 1). Although our sample size of patients was quite homogeneous, a high level of interindividual variability was identified in Vd and CL (Table 3). In general, there are high-level interindividual and intraindividual variabilities in linezolid PK, and the most frequently retained covariates in the PopPK models are age, body weight, creatinine clearance, and liver transaminase levels [28,37,38]. The estimated Vd (Table 3) is similar to the values found in the non-ECMO patients, and this corresponds to a total body fluid of 40–50 L [28,39,40,41], despite the fact that vv ECMO may increase the distribution of lipophilic drugs [10]. It has been previously observed that there is no difference in this PK parameter between critically ill non-ECMO patients and healthy volunteers, as the moderate lipophilic nature of linezolid is not affected by the increased extracellular fluid in critically ill patients [38]. Nevertheless, the estimated interindividual variability in Vd is 36.3% (relative standard error 19%) (Table 3), which captures the variations in the linezolid distribution characteristics among our patients. No statistically significant associations between the PK and vv ECMO parameters on the day of PK sampling (e.g., centrifugal pump rate) were identified, although this may be due to the small sample size. The estimated linezolid CL of 5.9 L/h has a good relationship with the other published data, including critically ill patients [28,38,39,40,41]. However, a wide range of linezolid CL values has been reported in regards to PopPK models, ranging from 2.5 to 11.2 L/h [28]. Our result (Table 3) suggests somewhat similar values compared with the median CL of 6.2 L/h in a typical non-ECMO patient of 70 kg and creatinine clearance rate of 80 mL/min, as reported in a review by Bandín-Vilaret et al. [28]. Then again, the large variability in CL among the different subgroups of patients limits the possibility of definitively positioning our results in comparison with others’. The previous publications have yielded conflicting findings regarding the influence of ECMO on the drugs’ PK [11]. Our estimated linezolid CL is lower than the CL based on infusion rate of 1800 mg/day and a mean serum concentration of 8.6 µg/mL in the vv ECMO patients, as per Kühn et al. [27]. However, based on the available data, it appears that ECMO probably has a less-significant impact on PK than the critical illness itself does [11].

Linezolid is a time-dependent antibiotic, with reported PK/PD targets of 85%T*f*>MIC, *f*AUC_24_/MIC of 80–120 [17,42]. For both the linezolid dosing regimens, 90% probabilities of both the *f*AUC_24_/MIC targets are attained when MIC ≤ 1 mg/L (Figure 5). Our result confirms the previous findings of De Rosa et al. in three ECMO patients on linezolid 600 mg every 12 h, where an AUC_24_/MIC ratio ≥ 80 was reached for MIC ≤ 1 mg/L [25]. However, for an MIC of 2 mg/L, *f*AUC_24_/MIC ≥ 80 was achieved in 87.44% and 29.54% of the patients following linezolid 600 mg IV every 8 h and 12 h, respectively. Moreover, great differences in the probability of 85%T*f*>MIC target attainment were observed between the two dosing regimens of linezolid. In other words, linezolid 600 mg IV every 8 h is predicted to achieve greater than or equal to 85%T*f*>MIC in 91.54% of the patients with CARDS on ECMO compared to only 67.86% of the patients on linezolid 600 mg every 12 h for an MIC value of 2 mg/L. As shown in Figure 5, an 85%T*f*>MIC PTA of 90% will be reached for MIC ≤ 2 mg/L after administering linezolid every 8 h, whereas for the 12-h interval, the same will be achieved for MICs ≤ 0.5 mg/L. As confirmed in El-Gaml et al. [29], reaching 85%T*f*>MIC is more probable with the continuous infusion of linezolid rather than intermittent administration. Therefore, our result confirms that shortening the time between doses from 12 to 8 h will substantially increase the probability of reaching 85%T*f*>MIC at an MIC of 2 mg/L. Our findings support the expert panel recommendations for linezolid dosing in ECMO patients when the MIC = 2 mg/L [24].

The general CFRs of the critically ill vv ECMO patients receiving two different dosing regimens of linezolid were estimated using EUCAST MIC distributions for *Enterococcus faecium*, *Staphylococcus aureus*, and *Streptococcus pneumoniae* [33]. The CFRs greatly varied among the PK/PD targets, pathogens, and dosing regimens (Figure 6). Summarizing the estimated CFRs, the target value of 90% is possible to reach with 600 mg every 8 h for each of the selected strains and 85%T*f*>MIC as a PK/PD target. In each of the tested scenarios, the CFR was higher with more intensive linezolid dosing (Figure 6); hence, this supports the empiric dosing of 600 mg every 8 h.

Several limitations should be considered for this study. First, it was designed as a single-center study with a small number of patients. The sample size was determined by the profoundly specialized and rare CARDS patient population on vv ECMO and linezolid dosing regimen of 600 mg every 8 h, together with our stringent inclusion/exclusion criteria. These criteria prevented us from describing the sources of the interindividual variability, and hence, none of the available covariates showed a significant effect on the linezolid PK parameters. We aimed to prioritize the quality of the data and the quantity of PK samples per patient. To the best of our knowledge, rich blood sampling has not been previously performed on vv ECMO patients after 600 mg every 8 h. It should be emphasized that this research did not include vv ECMO patients who were on renal replacement therapy, so the results do not account for this vulnerable patient group. In our study, the effect of the vv ECMO circuit could not be studied on PK linezolid, as all the patients were on vv ECMO support. Finally, linezolid was empirically introduced to address the pneumonia bacterial infections without the availability of microbiological data or MIC values. Unfortunately, all the patients included in our study ultimately succumbed to the severe complications of COVID-19, so a comparison between the patients’ outcomes and PTA or safety was not possible. Given the urgency of the situation and the evolving nature of the pandemic, this was a common practice during this time, where immediate intervention was prioritized to manage the infection.

## 5. Conclusions

This study characterized the concentration–time profile of linezolid after 30-min infusions of 600 mg every 8 h and established a PopPK model for this drug in patients with CARDS on vv ECMO therapy. This dosing regimen of linezolid was predicted to achieve greater than or equal to 85%T*f*>MIC in at least 90% of patients with CARDS on vv ECMO compared to only approximately two thirds of the patients on linezolid 600 mg every 12 h at an MIC of 2 mg/L. In addition, for the same MIC value, *f*AUC_24_/MIC ≥ 80 was achieved in almost three times the number of patients following linezolid 600 mg IV every 8 h versus 12 h. Nevertheless, the safety aspects (in particular, thrombocytopenia) should be carefully balanced against linezolid dosing, and consequently, monitoring is essential. In conclusion, our study suggests that 600 mg of linezolid administered every 8 h showed an advantage over 12-h dosing among the patients with CARDS on vv ECMO therapy when the MIC was equal or greater than 2 mg/L, or when empiric therapy was initiated.

## Figures and Tables

**Table 1:** Demographics and clinical characteristics of patients.

Characteristic	Median (Range/Percentage)	Interquartile Range
ICU admission		
Age (years)	40 (30–62)	30.4–59.8
Male (number)	5 (55.6%)	
BMI (kg/m^2^)	27.7 (23.5–39.2)	23.7–38.28
SAPS II	21 (13–36)	13.2–35.2
P/F ratio < 100	9 (100%)	
Murray score	3 (3–3.8)	3–3.55
Plasma creatinine concentration (µmol/L)	70 (53–349)	54.4–299
Urea (mmol/L)	5.5 (4.5–36.3)	4.52–30.88
Bilirubin (µmol/L)	9.9 (3.9–25.7)	4.34–24.3
Albumin (g/L)	32 (30–45)	30–43.4
Total protein (g/L)	60 (53–66)	53.4–65.8
D-dimer (mg/L)	1.1 (0.54–49.32)	0.546–40.75
On sampling day		
ECMO flow rate (L/min)	4.6 (3.4–6.5)	3.44–6.334
ECMO centrifugal pump speed (rmp)	3500 (2900–4300)	2920–4240
ECMO SF (L/min)	5 (4.5–8)	4.6–7.8
ECMO duration (days)	6 (1.75–17)	1.86–16.25
SOFA	10 (7–20)	7.2–18.4
Plasma creatinine concentration (µmol/L)	55 (34–201)	37.6–187.6
Urea (mmol/L)	7.2 (4.5–19.9)	4.72–19.36
Bilirubin (µmol/L)	37.4 (13.9–156.3)	14.3–148.44
Albumin (g/L)	38 (30–46)	30.2–45.4
Total protein (g/L)	51 (47–65)	47–64.6
24 h fluid balance (mL)	210 (−9475–5190)	−8190–4772

BMI, body mass index; SAPS II, simplified acute physiology score (II); P/F, arterial oxygen partial pressure (“P”) divided by the fractional inspired oxygen (“F”); ECMO extracorporeal membrane oxygenation; RMP, rotation per minute; ECMO SF, extracorporeal membrane oxygenation sweep gas flow; SOFA, sequential organ failure assessment.

**Table 2:** Individual pharmacokinetic (PK) characteristics of patients.

Patient ID	Peak Concentration (μg/mL)	Trough Concentration (μg/mL)	AUC_24_ (μg·h/mL)	Half-Life (h)
1	20.8	8.5	312.40	5.46
2	23.5	10.3	340.89	6.23
4	25.0	1.4	207.54	1.78
6	23.5	11.7	425.36	10.83
7	21.2	7.2	303.57	4.54
8	22.1	2.5	215.01	2.19
9	22.3	4.0	254.33	3.12
10	20.8	9.7	327.02	6.14
11	25.0	14.8	434.67	11.12

AUC_24_, daily area under the curve.

**Table 3:** Estimated population pharmacokinetic (PK) parameters of linezolid.

Parameter	Estimated Value	Bootstrap 95% Confidence Interval
Volume of distribution (L)	41.1	30.57–51.77
Clearance (L/h)	5.9	4.98–7.10
Proportional residual error	0.114	0.079–0.148
Interindividual variability in volume of distribution (%)	36.3	16.11–44.96
Interindividual variability in clearance (%)	24.8	13.03–31.25
Covariance of volume of distribution-clearance	−0.0901	−0.1353–−0.0212

## Data Availability

Data is unavailable due to privacy or ethical restrictions.
